# (*E*)-2-Meth­oxy-4-(3-oxobut-1-en­yl)phenyl acetate

**DOI:** 10.1107/S1600536808021417

**Published:** 2008-07-16

**Authors:** Huoyun Zhang, Shuqin Li, Xiaopeng Shi

**Affiliations:** a Department of Chemistry and Biology, Xiangfan University, Xiangfan 441053, People’s Republic of China

## Abstract

The title compound, C_13_H_14_O_4_, belongs to the class of α,β-unsaturated ketones, which have potential bactericidal, fungicidal, anti­tumor and anti-inflammatory properties. The C atoms and attached H atoms of the ethenyl part of the title mol­ecule are disordered over two orientations with refined occupancies of 0.583 (7) and 0.417 (3). Mol­ecules are connected by two inter­molecular C—H⋯O inter­actions, forming a dimer with 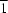 symmetry.

## Related literature

For related literature, see: Steiner *et al.* (1998 ▶); Kuo *et al.* (2005 ▶); Buszek *et al.* (2007 ▶); Yarishkin *et al.* (2008 ▶); Etter *et al.* (1990 ▶).
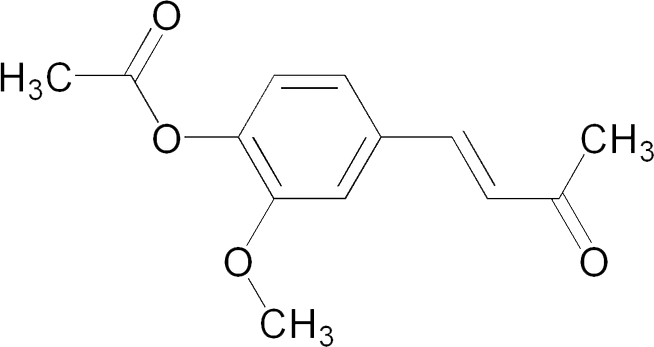


## Experimental

### 

#### Crystal data

C_13_H_14_O_4_
*M* *_r_* = 234.24
Triclinic, 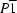

*a* = 6.2534 (5) Å
*b* = 7.5797 (5) Å
*c* = 13.9718 (8) Å
α = 96.611 (2)°
β = 91.487 (2)°
γ = 110.599 (2)°
*V* = 614.21 (7) Å^3^
*Z* = 2
Mo *K*α radiation
μ = 0.09 mm^−1^
*T* = 299 (2) K
0.30 × 0.23 × 0.20 mm

#### Data collection

Bruker SMART 4K CCD area-detector diffractometer
Absorption correction: multi-scan (*SADABS*; Sheldrick, 1997 ▶) *T*_min_ = 0.981, *T*_max_ = 0.989
3984 measured reflections
2381 independent reflections
1423 reflections with *I* > 2σ(*I*)
*R*_int_ = 0.049

#### Refinement

*R*[*F* ^2^ > 2σ(*F* ^2^)] = 0.066
*wR*(*F* ^2^) = 0.220
*S* = 1.10
2381 reflections
173 parameters
14 restraints
H-atom parameters constrained
Δρ_max_ = 0.23 e Å^−3^
Δρ_min_ = −0.47 e Å^−3^

### 

Data collection: *SMART* (Bruker, 1997 ▶); cell refinement: *SAINT* (Bruker, 1999 ▶); data reduction: *SAINT*; program(s) used to solve structure: *SHELXS97* (Sheldrick, 2008 ▶); program(s) used to refine structure: *SHELXL97* (Sheldrick, 2008 ▶); molecular graphics: *SHELXTL* (Sheldrick, 2008 ▶); software used to prepare material for publication: *SHELXTL*.

## Supplementary Material

Crystal structure: contains datablocks I, global. DOI: 10.1107/S1600536808021417/fb2101sup1.cif

Structure factors: contains datablocks I. DOI: 10.1107/S1600536808021417/fb2101Isup2.hkl

Additional supplementary materials:  crystallographic information; 3D view; checkCIF report

## Figures and Tables

**Table 1:** Hydrogen-bond geometry (Å, °)

*D*—H⋯*A*	*D*—H	H⋯*A*	*D*⋯*A*	*D*—H⋯*A*
C2—H2⋯O1^i^	0.93	2.58	3.507 (4)	172

Symmetry code: (i) 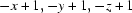.

**Fig. 1.**
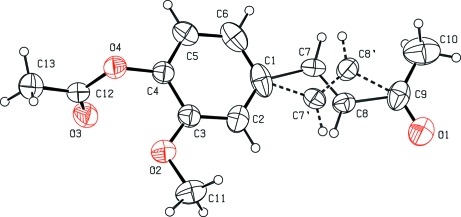
View of the title molecule disordered over two positions showing the atom-labelling scheme. The displacement ellipsoids are drawn at the 30% probability level. The H atoms are represented by spheres of arbitrary radius.

**Fig. 2.**
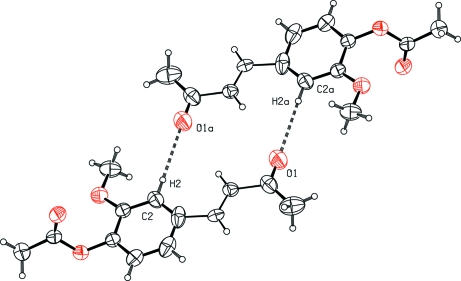
Intermolecular C—H···O interactions (dotted lines) in the title compound. [Symmetry code: 1 - x, 1 - y, 1 - z]

## supplementary crystallographic information

### Comment

The title compound belongs to α,β-unsaturated ketones having potential bactericidal, fungicidal, antitumor and antiinflammatory properties (Kuo *et al.*, 2005; Yarishkin *et al.*, 2008). Sythesis of α,β-unsaturated ketones is being extensively investigated (Buszek *et al.*, 2007). The molecular structure is shown in Fig. 1. The molecules form dimers by a pair of intermolecular C—H···O hydrogen bonds (Steiner *et al.*, 1998) forming a graph-set R^2^_2_(14) (Etter *et al.*, 1990).

### Experimental

(*E*)-4-(4-Hydroxy-3-methoxyphenyl)but-3-en-2-one (1.92 g, 0.01 mol) and Et_3_N (1.21 g, 0.012 mol) was dissolved in dry CH_2_Cl_2_ (50 ml). Acetyl chloride (1.02 g, 0.012 mol) was slowly added (ten minutes) to this solution by a syringe. The mixture was stirred at room temperature until the disappearance of ketone (monitored by thin layer chromatography). Then the mixture was poured into 50 ml brine and extracted with CH_2_Cl_2_ (40 ml). The organic layer was combined and dried over anhydrous Na_2_SO_4_. Removal of the solvent under reduced pressure and purification of the residue by recrystallization gave the title compound (2.1 g, yield 90%). The colourless crystals (average dimensions 0.3 mm × 0.2 mm × 0.2 mm) suitable for X-ray data collection were obtained by slow evaporation of a CH_2_Cl_2_ and MeOH solution in a ratio 4:1 at 293 K.

### Refinement

The difference electron density map has shown that the structure is disordered over two orientations in the E-ethen-1,2-yl group (C7 and C8 atoms). The H atoms could have been distinguished in the difference electron density maps, even in the disordered parts. The disordered parts were assumed to have the same geometry. The applied constraints: The sum of the occupancies of the disordered parts equaled to 1; the methyl as well as the aryl and the ethenyl H atoms were refined in idealized geometry with distances equal to 0.96, 0.93 and 0.93 Å, respectively. *U*_iso_=1.2*U*_eq_(C_aryl_/C_ethenyl_) and *U*_iso_=1.5*U*_eq_(C_methyl_). As to the restraints the pairs of the distances C8-C9 and C8-C9'; C1-C7 and C1-C7'; C7-C8 and C7-C8' were set to be as close possible by the command SADI with the effective standard uncertainty set to 0.001. The displacement parameters of C1, C7, C7', C8, C8', C9 were subjected to the restraint DELU 0.001 001.

From the refinement have been omitted diffractions 0 0 1; -2 0 2; -2 0 4, -1 0 2, -1 0 1 that did not match the model.

The refined occupational parameters of the disordered groups C7(H7)-C8(H8) and C7'(H7')-C8'(H8') converged to 0.568 (5) and 0.432 (5), respectively.

### Crystal data

**Table:**

C_13_H_14_O_4_	*Z* = 2
*M**_r_* = 234.24	*F*_000_ = 248
Triclinic, *P*1	*D*_x_ = 1.267 Mg m^−^^3^
Hall symbol: -P 1	Mo *K*α radiation λ = 0.71073 Å
*a* = 6.2534 (5) Å	Cell parameters from 1174 reflections
*b* = 7.5797 (5) Å	θ = 3.8–27.7º
*c* = 13.9718 (8) Å	µ = 0.09 mm^−^^1^
α = 96.611 (2)º	*T* = 299 (2) K
β = 91.487 (2)º	Block, colourless
γ = 110.599 (2)º	0.30 × 0.23 × 0.20 mm
*V* = 614.21 (7) Å^3^	

### Data collection

**Table:**

Bruker SMART 4K CCD area-detector diffractometer	2381 independent reflections
Radiation source: fine-focus sealed tube	1423 reflections with *I* > 2σ(*I*)
Monochromator: graphite	*R*_int_ = 0.049
*T* = 299(2) K	θ_max_ = 26.0º
φ and ω scans	θ_min_ = 2.9º
Absorption correction: multi-scan(SADABS; Sheldrick, 1997)	*h* = −7→7
*T*_min_ = 0.981, *T*_max_ = 0.989	*k* = −8→9
3984 measured reflections	*l* = −10→17

### Refinement

**Table:**

Refinement on *F*^2^	Primary atom site location: structure-invariant direct methods
Least-squares matrix: full	Secondary atom site location: difference Fourier map
*R*[*F*^2^ > 2σ(*F*^2^)] = 0.066	Hydrogen site location: difference Fourier map
*wR*(*F*^2^) = 0.220	H-atom parameters constrained
*S* = 1.10	*w* = 1/[σ^2^(*F*_o_^2^) + (0.1082*P*)^2^ + 0.0333*P*] where *P* = (*F*_o_^2^ + 2*F*_c_^2^)/3
2381 reflections	(Δ/σ)_max_ = 0.001
173 parameters	Δρ_max_ = 0.23 e Å^−^^3^
14 restraints	Δρ_min_ = −0.47 e Å^−^^3^
61 constraints	Extinction correction: none

### Special details

**Table:**

Geometry. All e.s.d.'s (except the e.s.d. in the dihedral angle between two l.s. planes) are estimated using the full covariance matrix. The cell e.s.d.'s are taken into account individually in the estimation of e.s.d.'s in distances, angles and torsion angles; correlations between e.s.d.'s in cell parameters are only used when they are defined by crystal symmetry. An approximate (isotropic) treatment of cell e.s.d.'s is used for estimating e.s.d.'s involving l.s. planes.
Refinement. Refinement of *F*^2^ against ALL reflections. The weighted *R*-factor *wR* and goodness of fit *S* are based on *F*^2^, conventional *R*-factors *R* are based on *F*, with *F* set to zero for negative *F*^2^. The threshold expression of *F*^2^ > σ(*F*^2^) is used only for calculating *R*-factors(gt) *etc*. and is not relevant to the choice of reflections for refinement. *R*-factors based on *F*^2^ are statistically about twice as large as those based on *F*, and *R*- factors based on ALL data will be even larger.

### Fractional atomic coordinates and isotropic or equivalent isotropic displacement parameters (Å^2^)

**Table:**

	*x*	*y*	*z*	*U*_iso_*/*U*_eq_	Occ. (<1)
C1	0.7293 (5)	0.3948 (4)	0.2545 (3)	0.0882 (10)	
C2	0.9194 (5)	0.5292 (4)	0.3052 (2)	0.0738 (8)	
H2	0.9074	0.5763	0.3685	0.089*	
C3	1.1270 (4)	0.5962 (3)	0.26515 (19)	0.0624 (7)	
C4	1.1411 (4)	0.5259 (3)	0.16982 (19)	0.0609 (7)	
C5	0.9549 (5)	0.3919 (4)	0.1173 (2)	0.0768 (8)	
H5	0.9673	0.3460	0.0538	0.092*	
C6	0.7504 (5)	0.3261 (4)	0.1588 (3)	0.0937 (11)	
H6	0.6241	0.2349	0.1232	0.112*	
C7	0.4817 (6)	0.2784 (6)	0.2726 (3)	0.0590 (12)	0.568 (5)
H7	0.3809	0.1860	0.2267	0.071*	0.568 (5)
C8	0.4287 (6)	0.3213 (5)	0.3572 (2)	0.0590 (12)	0.568 (5)
H8	0.5350	0.4157	0.4006	0.071*	0.568 (5)
C7'	0.5396 (6)	0.3583 (5)	0.3256 (2)	0.0695 (19)	0.432 (5)
H7'	0.5659	0.4348	0.3849	0.083*	0.432 (5)
C8'	0.3469 (9)	0.2245 (8)	0.3042 (3)	0.0690 (17)	0.432 (5)
H8'	0.3087	0.1433	0.2462	0.083*	0.432 (5)
C9	0.1831 (5)	0.2135 (5)	0.3871 (3)	0.0823 (9)	
C10	−0.0103 (8)	0.0611 (5)	0.3285 (3)	0.1215 (14)	
H10A	−0.1400	0.0213	0.3668	0.182*	
H10B	0.0349	−0.0452	0.3082	0.182*	
H10C	−0.0506	0.1090	0.2728	0.182*	
C11	1.3290 (6)	0.7714 (5)	0.4125 (2)	0.0957 (10)	
H11A	1.2309	0.8425	0.4272	0.144*	
H11B	1.4830	0.8466	0.4371	0.144*	
H11C	1.2771	0.6568	0.4420	0.144*	
C12	1.4400 (5)	0.7588 (4)	0.10439 (18)	0.0678 (7)	
C13	1.6743 (5)	0.7974 (5)	0.0730 (2)	0.0937 (10)	
H13A	1.6724	0.6999	0.0222	0.141*	
H13B	1.7741	0.7986	0.1266	0.141*	
H13C	1.7286	0.9187	0.0497	0.141*	
O1	0.1650 (4)	0.2704 (4)	0.46647 (19)	0.1071 (8)	
O2	1.3230 (3)	0.7243 (3)	0.31006 (13)	0.0802 (6)	
O3	1.3305 (3)	0.8606 (3)	0.10693 (14)	0.0814 (6)	
O4	1.3533 (3)	0.5806 (2)	0.13013 (12)	0.0693 (6)	

### Atomic displacement parameters (Å^2^)

**Table:**

	*U*^11^	*U*^22^	*U*^33^	*U*^12^	*U*^13^	*U*^23^
C1	0.0607 (15)	0.0758 (16)	0.144 (2)	0.0288 (13)	0.0191 (15)	0.0587 (17)
C2	0.0849 (16)	0.0753 (14)	0.0869 (16)	0.0450 (13)	0.0302 (12)	0.0313 (12)
C3	0.0681 (13)	0.0545 (11)	0.0715 (14)	0.0262 (10)	0.0083 (11)	0.0124 (10)
C4	0.0657 (13)	0.0539 (11)	0.0710 (14)	0.0260 (10)	0.0114 (10)	0.0120 (10)
C5	0.0794 (16)	0.0613 (13)	0.0850 (16)	0.0210 (12)	−0.0046 (12)	0.0102 (11)
C6	0.0754 (17)	0.0681 (15)	0.127 (2)	0.0162 (13)	−0.0100 (16)	0.0285 (15)
C7	0.071 (3)	0.047 (2)	0.056 (2)	0.022 (2)	−0.007 (2)	−0.0067 (18)
C8	0.069 (4)	0.060 (3)	0.051 (3)	0.024 (3)	0.001 (2)	0.003 (2)
C8'	0.075 (4)	0.072 (4)	0.059 (4)	0.029 (3)	0.015 (3)	0.009 (3)
C7'	0.061 (4)	0.046 (3)	0.067 (5)	0.019 (3)	0.001 (3)	0.006 (3)
C9	0.0968 (19)	0.0915 (17)	0.0871 (18)	0.0544 (16)	0.0341 (15)	0.0406 (15)
C10	0.170 (3)	0.105 (2)	0.111 (2)	0.077 (2)	−0.014 (2)	0.0052 (17)
C11	0.118 (2)	0.1023 (18)	0.0702 (16)	0.0465 (16)	−0.0048 (13)	−0.0076 (13)
C12	0.0737 (14)	0.0587 (13)	0.0602 (13)	0.0164 (11)	0.0068 (10)	−0.0040 (9)
C13	0.0821 (17)	0.0900 (17)	0.1022 (19)	0.0205 (14)	0.0215 (13)	0.0031 (13)
O1	0.1010 (13)	0.1159 (14)	0.0938 (13)	0.0276 (11)	0.0235 (10)	0.0089 (11)
O2	0.0838 (11)	0.0797 (10)	0.0694 (10)	0.0189 (9)	0.0060 (7)	−0.0027 (7)
O3	0.0893 (11)	0.0624 (9)	0.0981 (12)	0.0285 (9)	0.0171 (8)	0.0156 (8)
O4	0.0753 (10)	0.0609 (9)	0.0759 (10)	0.0279 (7)	0.0172 (7)	0.0076 (7)

### Geometric parameters (Å, °)

**Table:**

C1—C2	1.375 (4)	C7'—H7'	0.9300
C1—C6	1.405 (5)	C8'—C9	1.556 (4)
C1—C7	1.533 (4)	C8'—H8'	0.9300
C1—C7'	1.538 (4)	C9—O1	1.166 (3)
C2—C3	1.377 (4)	C9—C10	1.488 (5)
C2—H2	0.9300	C10—H10A	0.9600
C3—O2	1.349 (3)	C10—H10B	0.9600
C3—C4	1.393 (3)	C10—H10C	0.9600
C4—C5	1.370 (4)	C11—O2	1.431 (3)
C4—O4	1.395 (3)	C11—H11A	0.9600
C5—C6	1.370 (4)	C11—H11B	0.9600
C5—H5	0.9300	C11—H11C	0.9600
C6—H6	0.9300	C12—O3	1.197 (3)
C7—C8	1.273 (3)	C12—O4	1.362 (3)
C7—H7	0.9300	C12—C13	1.479 (4)
C8—C9	1.559 (4)	C13—H13A	0.9600
C8—H8	0.9300	C13—H13B	0.9600
C7'—C8'	1.274 (4)	C13—H13C	0.9600
			
C2—C1—C6	118.2 (3)	C7'—C8'—H8'	124.2
C2—C1—C7	138.3 (3)	C9—C8'—H8'	124.2
C6—C1—C7	103.5 (3)	O1—C9—C10	121.7 (3)
C2—C1—C7'	105.1 (3)	O1—C9—C8'	145.9 (3)
C6—C1—C7'	136.7 (3)	C10—C9—C8'	92.4 (3)
C1—C2—C3	122.0 (3)	O1—C9—C8	109.7 (3)
C1—C2—H2	119.0	C10—C9—C8	128.6 (3)
C3—C2—H2	119.0	C9—C10—H10A	109.5
O2—C3—C2	126.0 (3)	C9—C10—H10B	109.5
O2—C3—C4	115.8 (2)	H10A—C10—H10B	109.5
C2—C3—C4	118.2 (3)	C9—C10—H10C	109.5
C5—C4—C3	121.3 (2)	H10A—C10—H10C	109.5
C5—C4—O4	119.3 (2)	H10B—C10—H10C	109.5
C3—C4—O4	119.2 (2)	O2—C11—H11A	109.5
C4—C5—C6	119.6 (3)	O2—C11—H11B	109.5
C4—C5—H5	120.2	H11A—C11—H11B	109.5
C6—C5—H5	120.2	O2—C11—H11C	109.5
C5—C6—C1	120.8 (3)	H11A—C11—H11C	109.5
C5—C6—H6	119.6	H11B—C11—H11C	109.5
C1—C6—H6	119.6	O3—C12—O4	121.7 (2)
C8—C7—C1	112.9 (2)	O3—C12—C13	128.1 (3)
C8—C7—H7	123.6	O4—C12—C13	110.2 (3)
C1—C7—H7	123.6	C12—C13—H13A	109.5
C7—C8—C9	119.1 (3)	C12—C13—H13B	109.5
C7—C8—H8	120.5	H13A—C13—H13B	109.5
C9—C8—H8	120.5	C12—C13—H13C	109.5
C8'—C7'—C1	120.7 (3)	H13A—C13—H13C	109.5
C8'—C7'—H7'	119.6	H13B—C13—H13C	109.5
C1—C7'—H7'	119.6	C3—O2—C11	117.5 (2)
C7'—C8'—C9	111.7 (2)	C12—O4—C4	118.03 (19)

### Hydrogen-bond geometry (Å, °)

**Table:**

*D*—H···*A*	*D*—H	H···*A*	*D*···*A*	*D*—H···*A*
C2—H2···O1^i^	0.93	2.58	3.507 (4)	172

Symmetry codes: (i) −*x*+1, −*y*+1, −*z*+1.
